# Prediction of Adverse Outcomes in De Novo Hypertensive Disorders of Pregnancy: Development and Validation of Maternal and Neonatal Prognostic Models

**DOI:** 10.3390/healthcare10112307

**Published:** 2022-11-18

**Authors:** Junjun Chen, Yuelong Ji, Tao Su, Ma Jin, Zhichao Yuan, Yuanzhou Peng, Shuang Zhou, Heling Bao, Shusheng Luo, Hui Wang, Jue Liu, Na Han, Hai-Jun Wang

**Affiliations:** 1 Department of Electrical and Computer Engineering, Whiting School of Engineering, Johns Hopkins University, Baltimore, MD 21218, USA; 2 National Health Commission Key Laboratory of Reproductive Health, Department of Maternal and Child Health, School of Public Health, Peking University, Beijing 100191, China; 3 Tongzhou Maternal and Child Health Care Hospital of Beijing, Beijing 101101, China; 4 Department of Epidemiology and Biostatistics, School of Public Health, Peking University, Beijing 100191, China

**Keywords:** hypertension in pregnancy, preeclampsia, mortality

## Abstract

Effectively identifying high-risk patients with de novo hypertensive disorder of pregnancy (HDP) is required to enable timely intervention and to reduce adverse maternal and perinatal outcomes. Electronic medical record of pregnant women with de novo HDP were extracted from a birth cohort in Beijing, China. The adverse outcomes included maternal and fetal morbidities, mortality, or any other adverse complications. A multitude of machine learning statistical methods were employed to develop two prediction models, one for maternal complications and the other for perinatal deteriorations. The maternal model using the random forest algorithm produced an AUC of 0.984 (95% CI (0.978, 0.991)). The strongest predictors variables selected by the model were platelet count, fetal head/abdominal circumference ratio, and gestational age at the diagnosis of de novo HDP; The perinatal model using the boosted tree algorithm yielded an AUC of 0.925 (95% CI (0.907, 0.945]). The strongest predictor variables chosen were gestational age at the diagnosis of de novo HDP, fetal femur length, and fetal head/abdominal circumference ratio. These prediction models can help identify de novo HDP patients at increased risk of complications who might need intense maternal or perinatal care.

## 1. Introduction

Hypertensive disorders of pregnancy (HDP) are common complications in pregnant women that cause maternal and fetal morbidity and mortality worldwide, accounting for approximately 14.0% of maternal deaths per year [1]. The International Society for the Study of Hypertension in Pregnancy (ISSHP) classifies HDP into four categories, of which gestational hypertension and pre-eclampsia are the two de novo subtypes (de novo HDP) that contribute to most cases of the disorders. De novo HDP are characterized by the presence of hypertension occurred after 20 weeks of gestation and may be accompanied with proteinuria (a symptom with high levels of protein in the urine, indicating impaired kidney function), plus other maternal organ dysfunctions [2]. Patients with de novo HDP can suffer serious adverse maternal and neonatal outcomes, including stroke, acute kidney injury, heart failure, fetal growth restriction, preterm delivery, and even death [3]. These clinical deteriorations would require prolonged hospitalization as well as considerable medical resources and attention such as transferring to intense care unit [4]. Early prediction of de novo HDP’s adverse outcomes therefore becomes crucial to the planning and the allocating of care, especially when given the scarcity of medical resources in low- and middle-income countries where the incidence rates are the highest [5,6,7]. For example, a 2011 survey revealed that HDP affected 5.22% of all pregnancies in China, where 86.49% of the cases were gestational hypertension or pre-eclampsia (i.e., de novo HDP) [8].

Although clinical predictors (such as chest pain/dyspnea, low platelet count, increased AST/ALT, creatinine >100 µM, diastolic BP > 110 mm Hg) are widely used by international clinical practice guidelines as criteria for predicting outcome and classifying severity in women with de novo HDP, they often lack the ability to accurately distinguish those at higher risk of developing maternal or perinatal complications [9,10,11,12,13]. To address this global challenge, multiple studies have developed various risk prediction models: The fullPIERS and the subsequent miniPIERS were the first models to predict adverse maternal outcomes for patients with pre-eclampsia in high-income and low-income countries [14,15]. However, these studies were carried out in mostly non-east-Asian participants, and external validation of fullPIERS in the Chinese population did not lead to desirable results [16].

Hence, considering that the performance of the prediction model depends heavily on the target subjects and setting, two recent studies were conducted at different centers in China [17,18]. Both prediction models achieved relatively high AUC: 0.822 (95% CI [0.796, 0.847]) and 0.867 (95% CI [0.844, 0.890]). While these two localized models filled the gap by identifying east Asian patients with only pre-eclampsia that might develop maternal deterioration, they missed another large de novo HDP population that have gestational hypertension and, additionally, neglected the substantial risks faced by the patients’ fetuses. The objective of our study was to develop and validate two predictive models for adverse maternal and neonatal outcomes, whereby healthcare providers can effectively make assessment and take intervention for patients with de novo HDP.

## 2. Materials and Methods

This study was reported in line with the Transparent Reporting of a Multivariable Prediction Model for Individual Prognosis or Diagnosis (TRIPOD) statement [19].

### 2.1. Study Design and Population

We established a retrospective 7-year birth cohort at Tongzhou Maternal and Child Health Care Hospital of Beijing, which is a tertiary obstetric center in Northern China. This study enrolled patients diagnosed with de novo HDP (gestational hypertension or/and pre-eclampsia) that were admitted to the hospital between January 2012 and December 2019. By collecting data of the aforementioned subjects, we developed and validated two prediction models for adverse maternal outcomes or severe neonatal complications in patients with de novo HDP. The proposed timing to apply these models is when a pregnant woman has been diagnosed with de novo HDP, typically between 33 and 39 weeks into gestation, and the clinician, through experience, perceives the need for further identifying the risk of developing adverse outcomes. The time period for prediction was from the initial diagnosis of de novo HDP to delivery and discharge within a week. Approval by the Institutional Review Board of Peking University Health Science Center was obtained for this study (No. IRB00001052-21023). Consent was acquired, and patient information was deidentified and anonymized to ensure confidentiality.

### 2.2. Data Collection

A group of postgraduate students, obstetric nurses, and the hospital’s data engineers were recruited and trained using established data extraction criteria. Electronic medical records were either automatically mass extracted from the system, or manually inputted by the investigators. Consistency check was carried out regularly throughout the data extraction to ensure validity.

### 2.3. Inclusion and Exclusion Criteria

This study included pregnant women who delivered at the Tongzhou Maternal and Child Health Care Hospital of Beijing between January 2012 and December 2019, and satisfied the following conditions: (1) ≥18 years old; (2) singleton gestation; (3) diagnosed with de novo HDP during hospitalization; (4) not yet diagnosed with the above mentioned morbidities after admission but met the 2018 the International Society for the Study of Hypertension in Pregnancy (ISSHP) guidelines [2] and the 2020 Chinese HDP clinical practice guidelines [20]. According to these guidelines, gestational hypertension was defined as de novo onset (after 20 weeks of gestation) of hypertension (systolic blood pressure ≥140 mm Hg, diastolic blood pressure ≥90 mm Hg, or both) without the presence of proteinuria or other end-organ dysfunction; pre-eclampsia can be defined as new hypertension arising 20 weeks of gestation with proteinuria, other biochemical/hematological abnormalities, or both.

Patients were excluded from the study if they were transferred to other hospitals, did not undergo any pregnancy termination throughout hospitalization, or encountered any adverse outcome of interest ahead of the collection of predictor data. If a pregnant woman had multiple prior pregnancies, only the latest pregnancy’s information was kept. Other previous pregnancies diagnosed with de novo HDP were considered as having past de novo HDP history, which would be used as a predictor variable.

### 2.4. Candidate Predictors

Predictability, reliability, and accessibility were the criteria for selecting candidate predictor variables. In addition to general characteristics such as demographics and the number of prenatal checkups, variables considered in modeling (Table S1) were extracted from past medical history, cardiorespiratory tests, hematological tests, renal tests, hepatic tests, and fetal ultrasound measurements, diagnosis records, operative records, and progress records. The earliest values after the diagnosis of de novo HDP were chosen because this window would allow sufficient time for intervention before the onset of adverse outcomes. If such variable of a certain record was missing, the closest measure taken within a week before the diagnosis would be used as a replacement. Based on common missing data handling approaches, predictor variables (except for those having high *p* values) whose missingness was greater than 10% were excluded in the modeling because if such missingness was kept, the analysis was likely to be biased [21]. Variables with less than 10% missing data were imputed using the k-nearest neighbor algorithm, which takes the average from k (number) nearest neighbors found in the training set to impute the missing values. Clinical diagnostic cutoff points were used to generate supplementary categorical variables (high, low, or normal) based on their continuous counterparts. The final working dataset for maternal adverse outcome included 77 candidate predictor variables in total: 45 continuous, 32 categorical; For adverse neonatal outcome, the final working dataset included 80 candidate predictor variables in total: 44 continuous, 36 categorical.

### 2.5. Study Outcomes

The selected adverse maternal and neonatal outcomes were based on previous studies [14,15,17,18,22], systemic reviews [23], international clinical practice guidelines [2,11,12], and Chinese guidelines [20]. The outcomes included maternal and neonatal morbidities, mortality, or any other adverse events containing severe cerebrovascular, cardiorespiratory, liver, hematological, and renal complications (Table 1). These outcomes were defined using the International Classification of Diseases 10th Revision (ICD-10) codes and intensive care unit (ICU) admission records (extracted from electronic medical records). Ten adverse maternal outcomes were determined by the following ICD-10 codes: HELLP syndrome (O14.2), eclampsia (O15), cerebrovascular complications (O99), placental abruption (O45), acute kidney injury (O90.4), pulmonary edema (J81), liver dysfunction (S36.1), disseminated intravascular coagulation (D65), death (O95, R96, R99), and ICU admission. Six adverse neonatal outcomes were identified, including preterm birth, fetal growth restriction or small for gestational age, neonatal ICU admission, low Apgar scores, and neonatal death.

### 2.6. Development and Validation of the Model

The candidate predictor variables were filtered using univariate analysis: t test or Wilcoxon rank sum test for continuous variables and Pearson’s chi-square test or Fisher’s exact test for categorical variables. Variables associated (*p* ≤ 0.1) with the outcomes were fed into a multitude of machine learning algorithms to identify the best predictive models for maternal or neonatal outcome. The most representative machine learning methods of each type were screened and evaluated, including random forest, C5.0, bagged CART, boosted trees, k-nearest neighbors, neural network, flexible discriminant analysis, boosted logistic regression, naïve Bayesian, single C5.0 tree, boosted generalized linear model, elastic net, partial least squares, nearest shrunken centroids, bagged MARS, and tree models from genetic algorithms [24].

Ten-time repeated 10-fold cross validation was performed to randomly create 100 combinations of training and testing sets to optimize the machine learning models without overfitting. The best predictive machine learning methods for adverse maternal and neonatal outcomes were chosen based on their AUC (Area under the receiver operating characteristic curve: a metric to evaluate model performance; the larger the area the better), sensitivity (the model’s ability to identify positive instances), and specificity (the model’s ability to detect negative instances).

Machine learning algorithm screening carries considerable weight in our study, as this process determines the final performance of the prediction models. Machine learning algorithms can be assessed based on a variety of metrics, such as interpretability, computation speed, ease/difficulty of feature selection, and robustness to predictor noise. Each algorithm has its advantages and disadvantages. Careful performance screening and evaluation must be performed to determine the most suitable machine learning method(s) for our models. Two machine learning methods, random forest and boosted tree, were identified to be the most suitable in the study population. The first chosen algorithm, random forest, is a widely used supervised algorithm that creates a “forest” by growing and combining multiple decision trees. The logic behind the algorithm is that multiple individual decision tree models can achieve better performance as a group than separately. The random forest algorithm is less prone to overfitting and can yield high accuracy. It is also robust to outliers and predictor noise, maintaining relatively stable performance even when new data points are introduced into the dataset because this may only affect one decision tree. However, the random forest algorithm can be complex and time consuming compared to other methods. The second selected algorithm, boosted tree, is similar to random forest by repeatedly fitting multiple decision trees to improve the accuracy of the model. It uses the boosting method that weighs the input data in subsequent trees. This algorithm is easy to tune and robust to noise but can take longer to compute.

Backward feature elimination/selection (a technique that includes all features in the model than remove those less statistically significant variables one by one) was applied to the two selected models, shrinking their most informative predictor number to a manageable size for better application in clinical practice. For comparison, two additional benchmark models for adverse maternal and neonatal outcomes were created using the best performing machine learning methods identified by this study and fit with predictor variables deemed important in the fullPIERS study. These fullPIERS-selected variables included gestational age, chest pain or dyspnea, platelet count, creatinine, and aspartate transaminase concentrations, and oxygen saturation. We did not include oxygen saturation due to high percentage of unavailability in our study population, which was also found prone to be missing in the fullPIERS study [14]. R (Version 4.1.0) was used for data extraction, model fitting, and statistical analyses.

## 3. Results

### 3.1. Cohort Characteristics

As shown in Table 2, between January 2012 and December 2019, 1829 patients diagnosed de novo HDP were identified from a birth cohort study at the Tongzhou Maternal and Child Health Care Hospital of Beijing. A total of 102 (5.58%) patients developed adverse maternal outcomes, and 306 (16.73%) developed adverse neonatal outcomes after their diagnosis of de novo HDP. For both adverse maternal outcomes and neonatal outcomes, maternal age (years), gestational age at diagnosis (weeks) showed significant statistical difference, whereas BMI was only significant for maternal outcomes, and Parity for neonatal outcomes. The number of perinatal checkups were not analyzed for association was because only the more severe condition would lead to the more checkups, but not vice versa. These characteristics therefore should not be used as predictors. Compared to patients who had no adverse maternal outcomes, patients with complications had an earlier diagnosis of de novo HDP, lower fetal abdominal circumference, greater umbilical artery flow of the fetus, less frequent ultrasound checks, higher creatine, increased uric acid, higher aspartate aminotransferase and higher glutamate transaminase, elevated lactate dehydrogenase, higher total bilirubin, increased blood glucose, higher creatine kinase isoenzyme, higher urea nitrogen and increased electrolyte level, and lower platelet volume and thrombocytosis. Participants who developed adverse neonatal outcomes had similar characteristics. They had lower gestational age at the diagnosis of de novo HDP, lower fetal abdominal circumference, increased fetal umbilical artery flow, and higher creatine and uric acid levels. They also had higher aspartate aminotransferase and glutamate transaminase, increased lactate dehydrogenase, and elevated creatine kinase. The major adverse maternal outcomes for our study were HELLP syndrome (50.00%) and placental abruption (44.12%) (Table 1). The primary adverse neonatal outcome was preterm birth (89.87%) (Table 1).

### 3.2. Model Development, Specification, and Performance

Predictor variables having potential association (*p* ≤ 0.1) with adverse maternal outcomes or adverse neonatal outcomes were fed into numerous representative machine learning algorithms (Tables S2 and S3). After 10 repeated 10-fold cross validation, the best performing method in terms of AUC for adverse maternal outcome was the random forest algorithm, followed by bagged CART, C5.0, neural network, k-nearest neighbor, etc. (Figure S1). The top 10 most informative predictor variables selected by the random forest method included platelet count, fetal head/abdominal circumference ratio, gestational age at diagnosis, low plateletcrit (categorical), plateletcrit, 24 h urine protein, creatinine, high serum chlorine (categorical), fetal femur length/abdominal circumference ratio, and prothrombin time (Figure S2). For adverse neonatal outcomes, the best performing method in terms of AUC was boosted tree, followed by random forest, C5.0, flexible discriminant analysis, and bagged CART (Figure S3). The top 10 most important variables chosen by the boosted tree method included gestational age at diagnosis, fetal femur length, fetal head/abdominal circumference ratio, fetal biparietal diameter, fetal head circumference, 24 h urine protein, abnormal fetal head circumference (categorical), umbilical artery blood flow, resistance index, and fetal pulsatility index (Figure S4). These respective predictors for both adverse maternal and neonatal outcomes consistently spanned machine learning models, indicating their strong predictive power (Table S4(1,2)).

Table 3 showed the performance for each model with each set of predictor variables using the aforementioned backward feature elimination method. In terms of adverse maternal outcome model (random forest), the model with all predictor variables had the highest AUC, sensitivity, and specificity. Starting from the top 10 variables, the AUC began to drop. It was noted that models with less than 7 of the most important variables begin to drop faster in terms of sensitivity. Therefore, the simplified maternal model must contain at least the top 7 most important predictor variables (Table 3 and Figure S5). Applying the same approach to the adverse neonatal outcome model (boosted tree), the model with all variables had the highest AUC, sensitivity, and specificity. Interestingly, unlike the maternal models, the neonatal AUC values did not follow a linear decreasing manner. The model with the top 6 most important predictor variables had the second highest AUC and sensitivity, which should then be chosen as the final simplified neonatal model (Table 3 and Figure S6).

**Figure 1:**
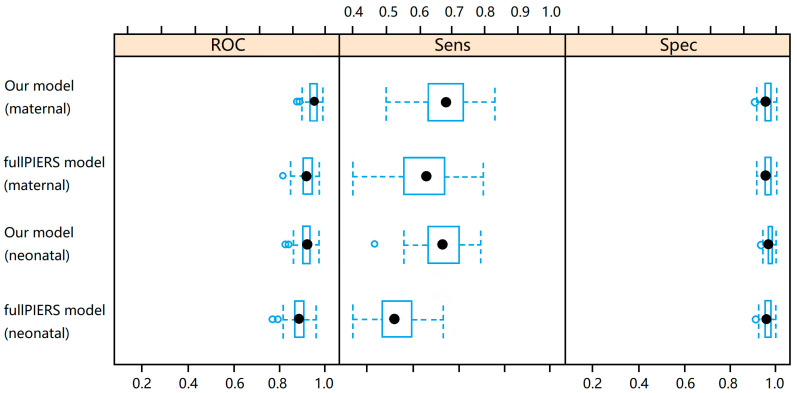
Performance comparison between our models and the full PIERS model for predicting adverse maternal and neonatal outcomes in our study population.

Based on the predictor variables selected in the fullPIERS model and the best performing machine learning algorithms selected by the two models developed in this study, the benchmark models using fullPIERS predictor variables and trained using the same dataset, however, were outperformed by our models (Table 4 and Figure 1). By comparison with the models developed in this study, although the benchmark models had similar AUCs of 0.942 (95% CI [0.930, 0.960]) vs. 0.965 (95% CI [0.953, 0.973]) for adverse maternal outcomes and 0.889 (95% CI [0.869, 0.911]) vs. 0.923 (95% CI [0.906, 0.937]) for neonatal outcomes, they had lower sensitivity (the ability to distinguish patients at risk): 0.623 (95% CI [0.554, 0.676]) for the benchmark maternal model vs. 0.686 (95% CI [0.629, 0.735]) for our maternal model; 0.323 (95% CI [0.267, 0.390]) for the benchmark neonatal model vs. 0.533 (95% CI [0.467, 0.600]) for our neonatal model.

## 4. Discussion

### Main Findings

We developed two risk prediction models using machine learning, aiming to identify patients with de novo HDP that might develop adverse maternal outcomes or severe neonatal deterioration. Our models predicted the risks of adverse maternal and neonatal outcomes as soon as the patients were diagnosed with de novo HDP. Using a variety of machine learning statistical methods, we identified informative and predictive risk factors, including those considered important in previous studies and additional features that pertained to our study population. In descending order of feature importance, predictor variables of the maternal model contained platelet count, fetal head/abdominal circumference ratio, gestational age at the diagnosis of de novo HDP, low plateletcrit (categorical), plateletcrit, 24 h urine protein, and creatinine; the predictor variables of the neonatal model comprised gestational age at the diagnosis of de novo HDP, fetal femur length, fetal head/abdominal circumference ratio, fetal biparietal diameter, fetal head circumference, and 24 h urine protein.

Except for gestational age, creatinine, and platelet count that were present in both our models and the fullPIERS model, the rest of the predictor variables selected by our models were different. For example, our models identified fetal head/abdominal circumference ratio as a crucial risk factor for predicting both adverse maternal and neonatal outcome. Besides this predictor variable from fetal ultrasound assessment, one more variable (fetal femur length/abdominal circumference ratio) from the same examination was also considered important in the maternal model. This finding indicates how demographic, geographic, and socio-economic differences in two study settings can play an essential role in model development, resulting in distinct sets of predictors in our study and the fullPIERS study. It also further emphasizes the importance of developing a tailored model that is more suitable for the local population and clinical practice, instead of deploying a model based on different population that might be underperforming in the new setting. These predictor variables deemed important by our models, in addition to carrying more predictive power in the Chinese population, they can also be easily accessed because the 3 types of tests that they were extracted from, comprehensive metabolic panel, complete blood count, and fetal ultrasound assessment, are commonly used in most hospitals across China. Our models should be able to facilitate and streamline management of health care given to pregnant women, in particular to those diagnosed with de novo HDP who can benefit the most from early perinatal intervention.

Apart from predictor variables such as platelet count and gestational age at the diagnosis of the disorder and creatinine, the models developed in this study also identified some novel predictor variables other than those of the other published models for adverse maternal outcome. Variables from the comprehensive metabolic panel and complete blood count constituted the majority of predictor variables for the maternal model, in addition to the fetal head/abdominal circumference ratio, which was identified as an important predictor in both our maternal and neonatal models as a clinical variable that was not traditionally considered. This latter variable, in conjunction with other predictor variables from the fetal ultrasound assessment, contributed to the most predictive variables selected by the neonatal model. In practice, if limited by the availability of clinical check-ups, the patients can possibly undergo a minimum of 3 types of tests (comprehensive metabolic panel, complete blood count, and fetal ultrasound assessment) to enable risk prediction for any adverse outcomes.

## 5. Conclusions

Between 2003 and 2009, hypertensive disorders of pregnancy (HDPs) accounted for 8–10% of all pregnancies and 14% of all maternal deaths globally [1]. In 2011, HDP affected 5.22% of all pregnancies in China, causing millions suffered from the disorders [8]. Multiple international and regional studies [14,15] were carried out to address this international challenge by developing risk assessment tools, which presented good prediction ability to identify patients at risk of adverse maternal outcome. We developed two risk prediction models using machine learning, aiming to identify patients with de novo HDP that might develop adverse maternal outcomes or severe neonatal complications. By comparison, our models not only concentrated on both the maternal and neonatal outcomes, but also outperformed the benchmark model by a significant margin. The risk prediction tool developed in this study can aid in identifying women at elevated risk of developing adverse maternal or neonatal outcomes, allowing providers to take timely prevention or intervention, thus elevating both health and financial burden.

Some strengths were presented in this study. First, we included a wide range of accessible clinical variables that fit the local clinical settings. Second, the gestational age of diagnosis for de novo HDP was extracted by a hybrid method, which guaranteed good data quality. We extracted the gestational age of the diagnosis from the free text of medical records using the regular expression matching method. Then, all the information was double checked by two independent researchers to examine abstraction accuracy. Third, compared to previous risk prediction models such as fullPIERS [14], miniPIERS [15], and the two other regional models developed for the Chinese population [17,18], our study also took tremendous consideration in modeling adverse neonatal outcomes, which had a relatively large case sample for analysis. Fourth, the important predictors identified in our models and their associated tests are readily available at most hospitals and can be acquired quickly at relatively low cost.

Meanwhile, there are still some limitations to our present study. First, our study collected data primarily from the suburban population in northern China (Beijing). The potential of our model’s generalizability needs more study to validate. Second, the units of diagnosis dates for de novo HDP are in weeks, which may lack temporal accuracy to build the model for predicting outcomes within several days. Third, our current models have not yet been externally validated. To address such limitations, it is necessary to establish a multi-center, multi-demographic collaboration with hospitals located in other parts of China, through which we can cross-validate our models and potentially develop a useful prediction tool with greater generalizability.

## Figures and Tables

**Table 1:** Occurrence of adverse maternal and neonatal outcomes by mortality/morbidity.

	Maternal Outcomes		Neonatal Outcomes
Total occurrence	102 (5.58%)	Total occurrence	306 (16.73%)
HELLP syndrome	51	Preterm birth	275
Eclampsia	3	Fetal growth restriction or small for gestational age	33
Cerebrovascular complications	0	Neonatal ICU admission	0
Placental abruption	45	Low Apgar scores (<7)	17
Acute kidney injury	0	Neonatal death	5
Pulmonary edema	6		
ICU admission	1		
Liver dysfunction	3		
Disseminated intravascular coagulation	2		
Maternal death	0		

**Table 2:** General characteristics of the study population.

	Adverse Maternal Outcomes			Adverse Neonatal Outcomes		
	No, *n* = 1 727	Yes, *n* = 102	*p*-Value	No, *n* = 1523	Yes, *n* = 306	*p*-Value
	Demographic characteristics					
Maternal age (years)	29.3 (4.3)	30.9 (4.5)	0.008	29.2 (4.3)	30.4 (4.5)	0.040
BMI	24.6 (4.0)	23.3 (3.5)	0.004	24.6 (4.1)	24.0 (3.4)	0.6
Gestational age at diagnosis (weeks)	36.5 (3.7)	33.7 (4.0)	<0.001	37.1 (3.5)	32.9 (3.2)	<0.001
Parity ≥1	425 (24.6)	30 (29.4)	0.4	367 (24.1)	89 (29.1)	0.036
Gravidity ≥ 1	824 (47.7)	50 (48.7)	>0.9	711 (46.7)	164 (53.6)	0.3
	Education level					
Low	276 (20.8)	20 (19.4)	0.2	319 (20.9)	59 (19.4)	0.7
Middle	608 (45.9)	41 (40.3)		695 (45.6)	138 (45.0)	
High	442 (33.3)	41 (40.3)		509 (33.4)	109 (35.6)	
	Occupation					
Worker/Farmer	69 (4.0)	3 (2.6)	0.8	61 (4.0)	12 (3.8)	0.5
Government Employee/technician	755 (43.7)	45 (44.7)		664 (43.6)	137 (44.7)	
Business/services	362 (20.9)	19 (18.4)		312 (20.5)	69 (22.6)	
Others	541 (31.3)	35 (34.2)		486 (31.9)	88 (28.8)	
	Number of checkups during perinatal period					
Perinatal checkups	9.6 (4.2)	7.8 (3.6)	NA	9.9 (4.1)	7.3 (3.3)	NA
Urine routine tests	38.4 (62.9)	33.9 (39.2)		35.3 (52.7)	52.3 (93.9)	
Comprehensive metabolic panel	5.2 (2.6)	6.9 (4.4)		4.9 (2.1)	7.1 (4.3)	
Blood coagulation factor	4.1 (2.2)	6.6 (4.2)		3.8 (1.9)	6.1 (3.7)	
Complete blood count	10.3 (3.3)	12.5 (4.9)		10.1 (3.1)	12.0 (4.7)	
Urine protein tests	28.2 (52.5)	25.5 (32.7)		25.4 (41.9)	41.3 (83.5)	
Fetal ultrasound tests	3.2 (1.2)	2.6 (0.9)		3.3 (1.2)	2.5 (1.0)	

Continuous variables: mean (standard deviation); Categorical variables: count (column percentage).

**Table 3:** The performance of selected models with different sets of top features based on backward feature elimination method.

Maternal Model				Neonatal Model			
Number of Features	AUC	Sens	Spec	Number of Features	AUC	Sens	Spec
All variables	0.986 (95% CI [0.978, 0.991])	0.783 (95% CI [0.735, 0.824])	0.994 (95% CI [0.988, 1.000])	All variables	0.930 (95% CI [0.907, 0.945])	0.548 (95% CI [0.484, 0.600])	0.980 (95% CI [0.974, 0.987])
Top 10	0.972 (95% CI [0.961, 0.981])	0.647 (95% CI [0.600, 0.714])	0.988 (95% CI [0.983, 0.994])	Top 6	0.923 (95% CI [0.906, 0.937])	0.533 (95% CI [0.467, 0.600])	0.974 (95% CI [0.972, 0.987])
Top 9	0.972 (95% CI [0.963, 0.979])	0.743 (95% CI [0.706, 0.794])	0.980 (95% CI [0.971, 0.988])	Top 7	0.922 (95% CI [0.905, 0.937])	0.541 (95% CI [0.484, 0.600])	0.974 (95% CI [0.967, 0.980])
Top 7	0.965 (95% CI [0.955, 0.974])	0.714 (95% CI [0.676, 0.765])	0.977 (95% CI [0.965, 0.983])	Top 9	0.922 (95% CI [0.902, 0.936])	0.525 (95% CI [0.467, 0.581])	0.974 (95% CI [0.972, 0.987])
Top 8	0.965 (95% CI [0.953, 0.973])	0.686 (95% CI [0.629, 0.735])	0.971 (95% CI [0.965, 0.983])	Top 8	0.922 (95% CI [0.902, 0.936])	0.525 (95% CI [0.467, 0.581])	0.974 (95% CI [0.972, 0.987])
Top 6	0.962 (95% CI [0.947, 0.970])	0.588 (95% CI [0.543, 0.629])	0.977 (95% CI [0.971, 0.983])	Top 10	0.921 (95% CI [0.903, 0.935])	0.516 (95% CI [0.452, 0.570])	0.980 (95% CI [0.974, 0.987])
Top 5	0.943 (95% CI [0.930, 0.958])	0.543 (95% CI [0.471, 0.591])	0.983 (95% CI [0.977, 0.988])	Top 5	0.906 (95% CI [0.890, 0.925])	0.452 (95% CI [0.387, 0.516])	0.961 (95% CI [0.948, 0.974])

**Table 4:** Performance comparison between our models and the benchmark models.

Models Developed in This Study				Benchmark Models			
	**AUC**	**Sens**	Spec		AUC	Sens	Spec
Maternal model	0.965 (95% CI [0.953, 0.973])	0.686 (95% CI [0.629, 0.735])	0.971 (95% CI [0.965, 0.983])	Maternal model	0.923 (95% CI [0.906, 0.937])	0.533 (95% CI [0.467, 0.600])	0.974 (95% CI [0.972, 0.987])
Neonatal model	0.942 (95% CI [0.930, 0.960])	0.623 (95% CI [0.555, 0.676])	0.971 (95% CI [0.965, 0.983])	Neonatal model	0.889 (95% CI [0.869, 0.911])	0.323 (95% CI [0.267, 0.390])	0.967 (95% CI [0.954, 0.980])

## Data Availability

Not applicable.

## Supplementary Materials

The following supporting information can be downloaded at: https://www.mdpi.com/article/10.3390/healthcare10112307/s1, Table S1. Predictor variables considered in modeling. Table S2. Univariate analyses of predictor variables (*p* ≤ 0.1) for adverse maternal outcomes. Table S3. Univariate analyses of predictor variables (*p* ≤ 0.1) for adverse neonatal outcomes. Table S4. (1,2) Top 5 predictors identified by machine learning models for both adverse and neonatal outcomes, respectively. Figure S1. Machine learning methods screened for modeling adverse maternal outcomes. Figure S2. Features selected by the random forest method (highest AUC). Figure S3. Machine learning methods screened for modeling adverse neonatal outcomes. Figure S4. Features selected by the boosted tree method (highest AUC). Figure S5. Backward feature elimination of the random forest model for adverse maternal outcome. Figure S6. Backward feature elimination of the boosted tree model for adverse neonatal outcomes.
