# Supplementary material for: Prediction of Adverse Outcomes in De Novo Hypertensive Disorders of Pregnancy: Development and Validation of Maternal and Neonatal Prognostic Models
Source: Healthcare (Basel). 2022 Nov 18;10(11):2307. doi: 10.3390/healthcare10112307 (PMC9690621; doi:10.3390/healthcare10112307)

Supplemental materials

Table S1. Predictor variables considered in modeling.

Table S2. Univariate analyses of predictor variables ($p \leq 0.1$) for adverse maternal outcomes.

Table S3. Univariate analyses of predictor variables ($p \leq 0.1$) for adverse neonatal outcomes.

Figure S1. Machine learning methods screened for modeling adverse maternal outcomes

Figure S2. Features selected by the random forest method (highest AUC)

Figure S3. Machine learning methods screened for modeling adverse neonatal outcomes

Figure S4. Features selected by the boosted tree method (highest AUC)

Figure S5. Backward feature elimination of the random forest model for adverse maternal outcome

Figure S6. Backward feature elimination of the boosted tree model for adverse neonatal outcomes

Table S1. Predictor variables (base) considered in modeling

**Demographic characteristics**	Thrombin time (sec)
Maternal age at pregnancy (years)	Lymphocyte level (%)
BMI	Activated partial thromboplastin time (sec)
Gestational age at the diagnosis of gestational hypertension or pre-eclampsia (weeks)	Triglyceride (mmol/L)
	Blood white blood cell count (x10^9/L)
Gravidity (n)	Monocyte ratio (%)
Parity (n)	Basophils ratio (%)
**Past medical history**	Carbon dioxide combining power (mmol/L)
Previous gestational hypertension (y/n)	Serum chlorine (mmol/L)
Chronic hypertension (y/n)	Serum calcium (mmol/L)
Previous gestational diabetes mellitus (y/n)	Serum sodium (mmol/L)
Chronic diabetes mellitus (y/n)	Serum potassium (mmol/L)
Renal disease (y/n)	Serum phosphorus (mmol/L)
**Symptoms**	**Renal tests**
Nausea or vomiting (y/n)	24 h urine protein (mg/24 h)
Frontal headache (y/n)	Creatine (umol/L)
Visual disturbance (y/n)	Uric acid (umol/L)
Chest pain or dyspnea (y/n)	Urobilinogen (umol/L)
**Cardiorespiratory signs**	Glucose in urine (mmol/L)
Diastolic blood pressure (mm Hg)	Urine white blood cell count (Leu/uL)
Systolic blood pressure (mm Hg)	Urine specific gravity
Mean arterial pressure (mm Hg)	Nitrites in urine (+/-)
Hydroxybutyrate dehydrogenase (U/L)	**Hepatic tests**
Creatine kinase (U/L)	Lactate dehydrogenase (U/L)
Creatine kinase isoenzymes (U/L)	Aspartate transaminase (U/L)
**Hematological tests**	Alanine transaminase (U/L)
Plateletcrit (%)	Gamma-glutamyl transferase (U/L)
Platelet count (x10^9/L)	Total bile acid (umol/L)
Prothrombin time (sec)	Bilirubin (umol/L)
Mean corpuscular hemoglobin (pg)	**Fetal assessments**
Blood urea nitrogen (mmol/L)	Abdominal circumference (cm)
Globulin (g/L)	Head circumference (cm)
Fibrinogen (g/L)	Biparietal diameter (cm)
Mean corpuscular hemoglobin concentration (g/L)	Femur length (cm)
Platelet-large cell ratio (%)	Resistance index
Mean platelet volume (fL)	Pulsatility index
Platelet distribution width (fL)	Umbilical artery flow

Table S2. Univariate analyses of predictor variables (p ≤ 0.1) for adverse maternal outcomes.

	OR (95% CI)	p value
**Demographic characteristics**		
Maternal age at pregnancy (years)	1.08 (1.03-1.13)	<0.001
Gestational age at the diagnosis of GH or PE (weeks)	0.87 (0.84-0.91)	<0.001
**Symptoms**		
Nausea or vomiting (y/n)	1.48 (0.91-2.31)	0.1
Chest pain or dyspnea (y/n)	2.65 (0.89-6.36)	0.048
**Cardiorespiratory signs**		
Hydroxybutyrate dehydrogenase (U/L)	1.00 (1.00-1.01)	<0.001
**Hematological tests**		
Plateletcrit (%)	1.00 (1.00-1.01)	<0.001
Platelet count (x10^9/L)	0.98 (0.98-0.99)	<0.001
Prothrombin time (sec)	0.45 (0.32-0.62)	<0.001
Mean corpuscular hemoglobin (pg)	1.32 (1.18-1.46)	<0.001
Blood urea nitrogen (mmol/L)	1.03 (1.02-1.04)	<0.001
Fibrinogen (g/L)	0.54 (0.40-0.74)	<0.001
Mean corpuscular hemoglobin concentration (g/L)	1.04 (1.02-1.06)	<0.001
Mean platelet volume (fL)	1.46 (1.24-1.72)	<0.001
Platelet-large cell ratio (%)	1.06 (1.03-1.08)	<0.001
Platelet distribution width (fL)	1.14 (1.05-1.25)	0.003
Thrombin time (sec)	1.08 (1.01-1.16)	0.022
Lymphocyte level (%)	1.05 (1.02-1.08)	0.002
Triglyceride (mmol/L)	0.90 (0.80-1.01)	0.1
**Renal tests**		
24 h urine protein (mg/24 h)	1.00 (1.00-1.00)	<0.001
Creatine (umol/L)	1.01 (1.00-1.02)	<0.001
Uric acid (umol/L)	1.00 (1.00-1.00)	<0.001

**Hepatic tests**		
Lactate dehydrogenase (U/L)	1.00 (1.00-1.01)	<0.001
Aspartate transaminase (U/L)	1.03 (1.02-1.04)	<0.001
Alanine transaminase (U/L)	1.03 (1.02-1.04)	<0.001
Gamma-glutamyl transferase (U/L)	1.01 (1.00-1.02)	0.008
Total bile acid (umol/L)	1.04 (1.02-1.07)	<0.001
**Fetal assessments**		
Abdominal circumference (cm)	0.86 (0.83-0.90)	<0.001
Head circumference (cm)	1.01 (1.00-1.02)	0.089
Biparietal diameter (cm)	0.59 (0.49-0.70)	<0.001
Femur length (cm)	0.53 (0.43-0.65)	<0.001
Resistance index	964 (42.0-22813)	<0.001
Umbilical artery flow	2.35 (1.65-3.28)	<0.001

OR = Odds Ratio, CI = Confidence Interval

Table S3. Univariate analyses of predictor variables (p ≤ 0.1) for adverse neonatal outcomes.

	OR (95% CI)	p value
**Demographic characteristics**		
Maternal age at pregnancy (years)	1.06 (1.03-1.09)	<0.001
Gestational age at the diagnosis of GH or PE (weeks)	0.78 (0.75-0.81)	<0.001
**Symptoms**		
Frontal headache (y/n)	2.97 (2.09-4.19)	<0.001
Chest pain or dyspnea (y/n)	4.21 (2.17-8.06)	<0.001
**Cardiorespiratory signs**		
Hydroxybutyrate dehydrogenase (U/L)	1.01 (1.00-1.01)	<0.001
Creatine kinase (U/L)	1.00 (1.00-1.00)	<0.045
Creatine kinase isoenzymes (U/L)	1.00 (1.00-1.01)	0.029
**Hematological tests**		
Plateletcrit (%)	0.11 (0.01-1.34)	0.086
Platelet count (x10^9/L)	1.00 (1.00-1.00)	0.082
Prothrombin time (sec)	0.61 (0.50-0.73)	<0.001
Mean corpuscular hemoglobin (pg)	1.19 (1.12-1.27)	<0.001
Blood urea nitrogen (mmol/L)	1.29 (1.21-1.37)	<0.001
Fibrinogen (g/L)	0.55 (0.45-0.66)	<0.001
Mean corpuscular hemoglobin concentration (g/L)	1.04 (1.02-1.05)	<0.001
Platelet distribution width (fL)	0.81 (0.71-0.91)	<0.001
Thrombin time (sec)	1.12 (1.07-1.17)	<0.001
Activated partial thromboplastin time (sec)	1.08 (1.03-1.14)	0.001
Serum chlorine (mmol/L)	1.00 (1.00-1.01)	0.003
Serum potassium (mmol/L)	1.11 (1.04-1.19)	0.002
**Renal tests**		
24 h urine protein (mg/24 h)	1.00 (1.00-1.00)	<0.001
Creatine (umol/L)	1.01 (1.01-1.02)	<0.001

Uric acid (umol/L)	1.00 (1.00-1.00)	<0.001
**Hepatic tests**		
Lactate dehydrogenase (U/L)	1.01 (1.00-1.01)	<0.001
Aspartate transaminase (U/L)	1.03 (1.02-1.04)	<0.001
Alanine transaminase (U/L)	1.03 (1.02-1.04)	<0.001
Gamma-glutamyl transferase (U/L)	1.02 (1.01-1.02)	<0.001
Total bile acid (umol/L)	1.11 (1.08-1.14)	<0.001
**Fetal assessments**		
Head circumference (cm)	0.92 (0.89-0.95)	<0.001
Biparietal diameter (cm)	0.37 (0.33-0.42)	<0.001
Femur length (cm)	0.28 (0.23-0.32)	<0.001
Umbilical artery flow	4.95 (3.74-6.64)	<0.001

OR = Odds Ratio, CI = Confidence Interval

Figure S1. Machine learning methods screened for modeling adverse maternal outcomes

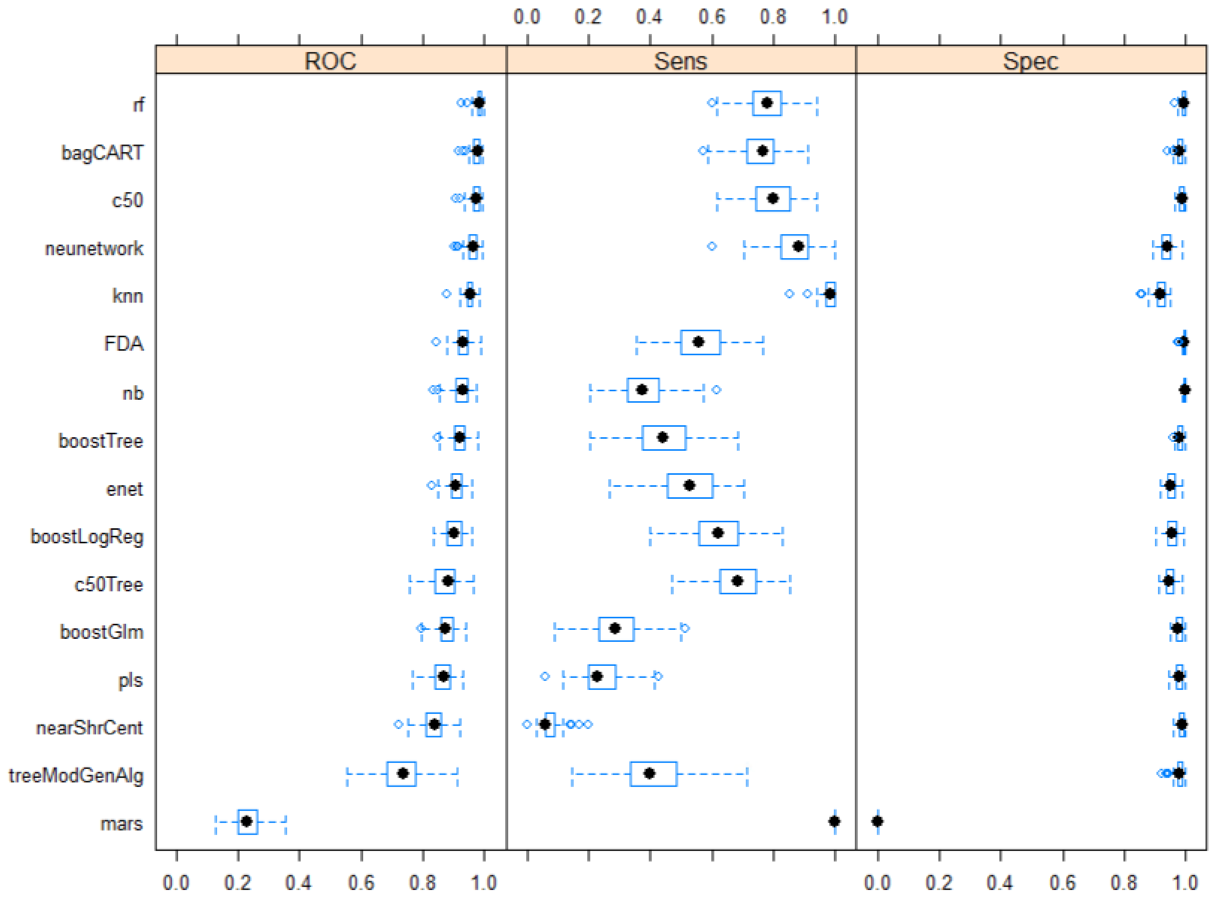

Figure S2. Features selected by the random forest method (highest AUC)

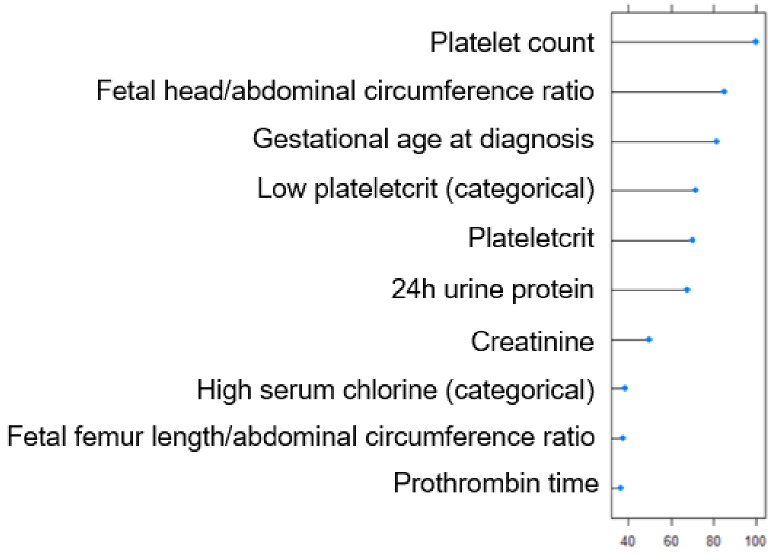

Figure S3. Machine learning methods screened for modeling adverse neonatal outcomes

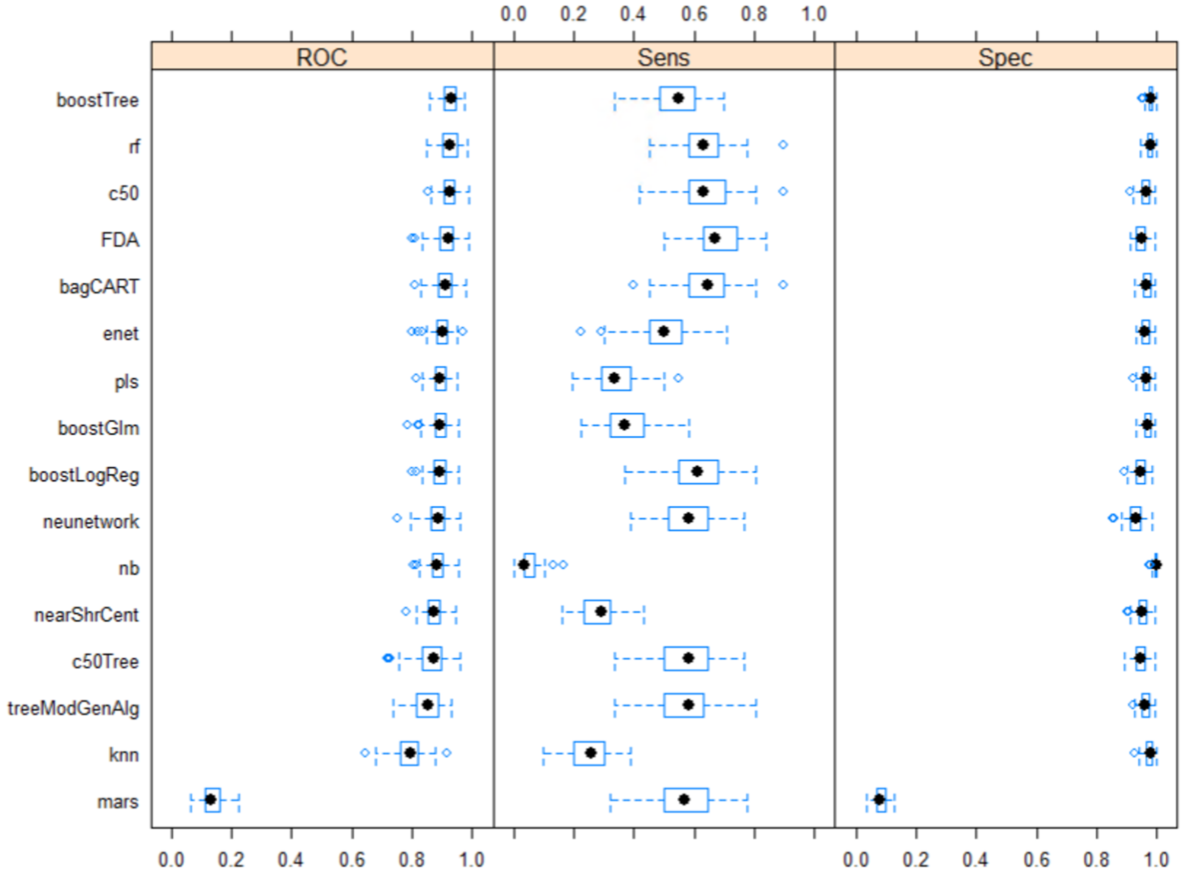

Figure S4. Features selected by the boosted tree method (highest AUC)

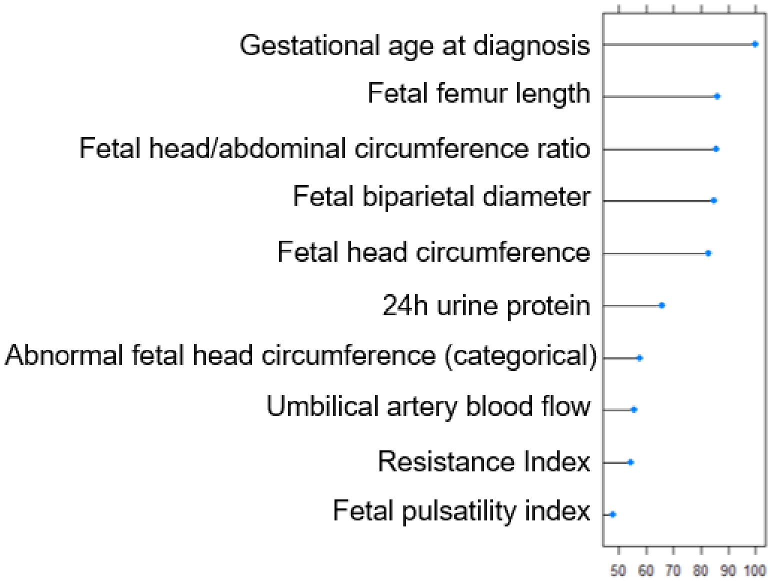

Figure S5. Backward feature elimination of the random forest model for adverse maternal outcome

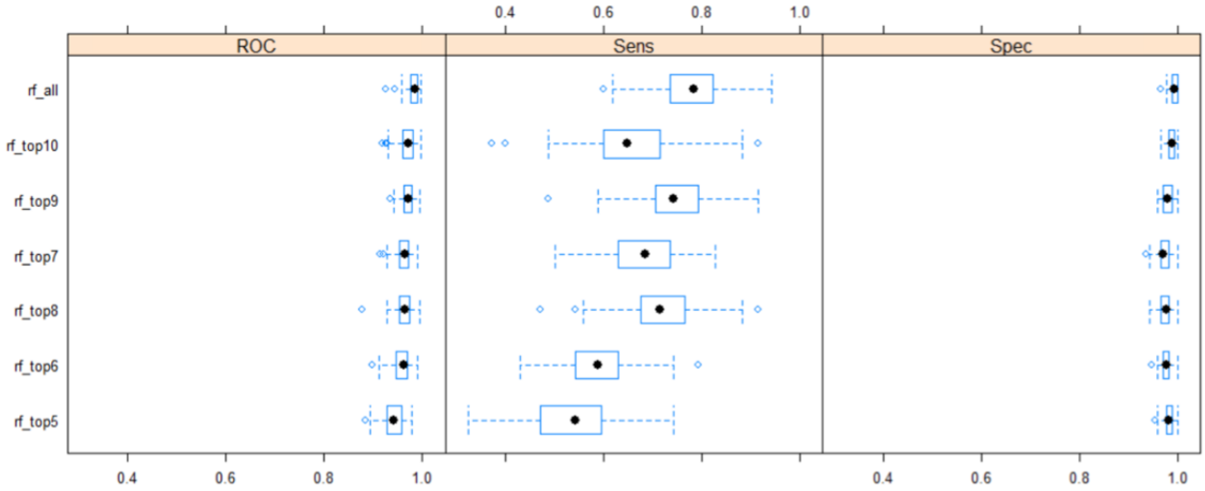

Figure S6. Backward feature elimination of the boosted tree model for adverse neonatal outcomes

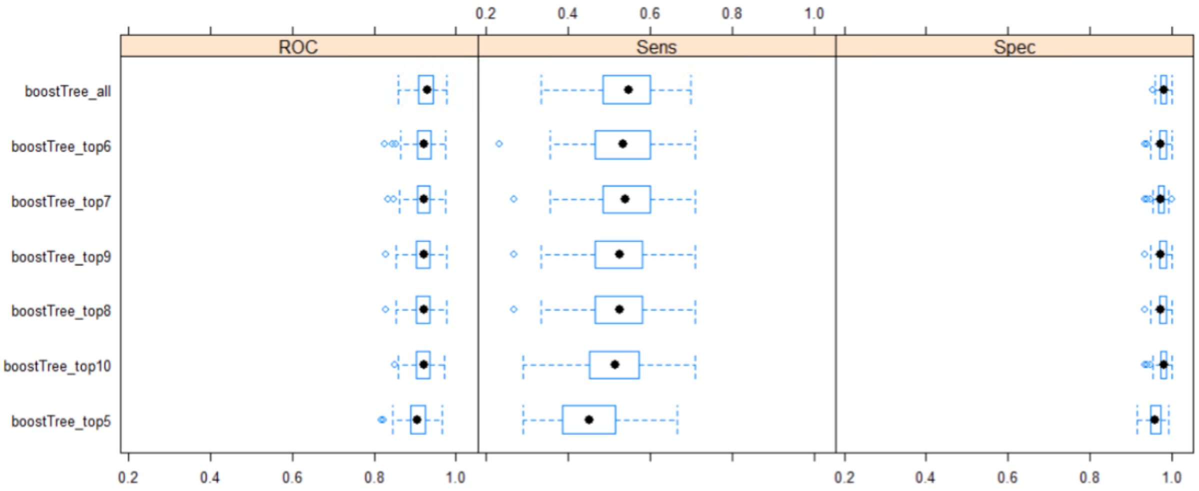

Supplement: Supplementary file 1 [file healthcare-10-02307-s001.zip › healthcare-1983327-supplementary.pdf]
